# Supplementary figures and images for: Phylogenomics of 8,839 Clostridioides difficile genomes reveals recombination-driven evolution and diversification of toxin A and B
Source: PLoS Pathog. 2020 Dec 28;16(12):e1009181. doi: 10.1371/journal.ppat.1009181 (PMC7853461; doi:10.1371/journal.ppat.1009181)

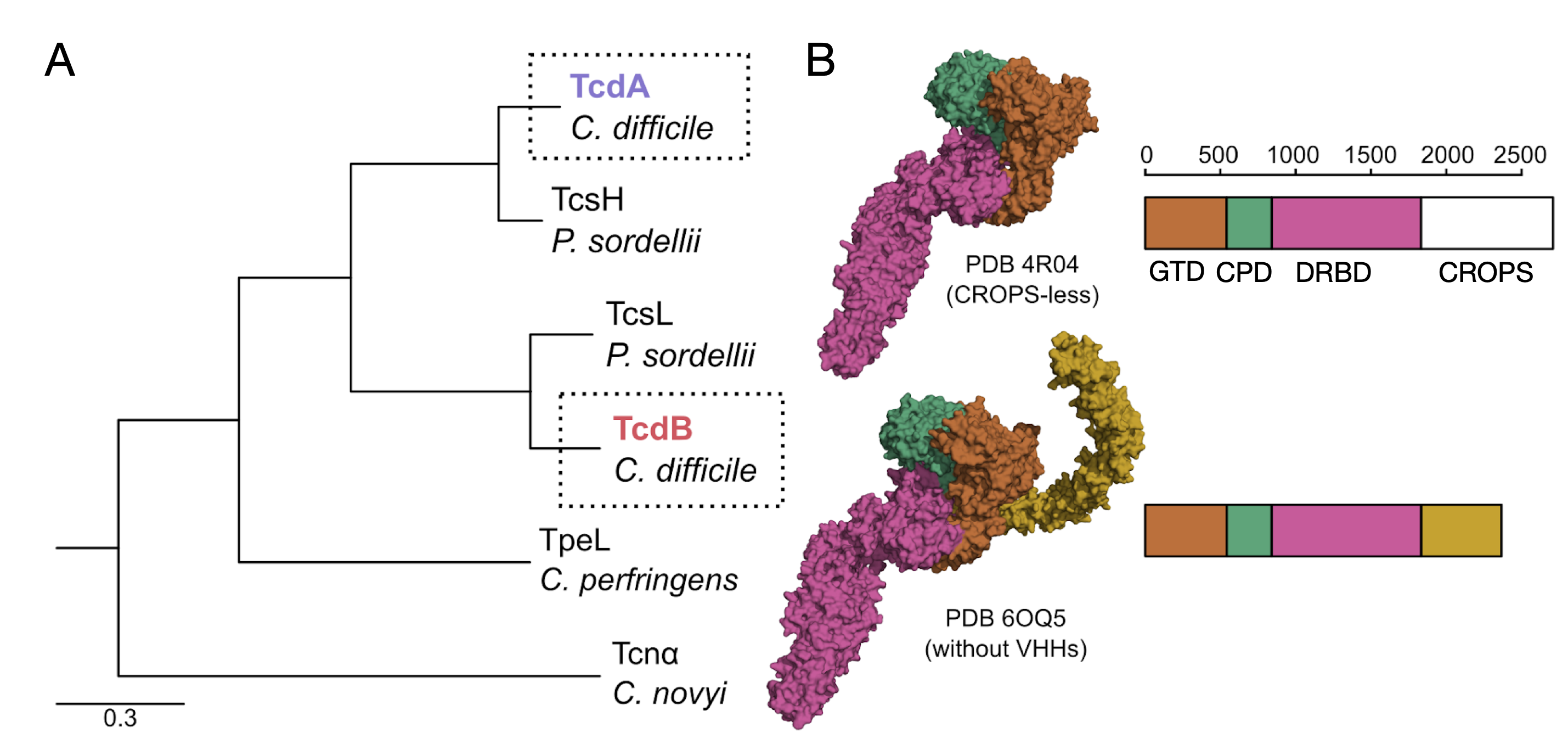

Supplement: S1 Fig — (A) The TcdA family forms a monophyletic clade with TcsH from Paeniclostridium sordellii as a sister phylogenetic lineage. Similarly, the TcdB family forms a monophyletic clade with TcsL from Paeniclostridium sordellii as a sister phylogenetic lineage. This implies a scenario whereby TcdA and TcdB evolved by an ancestral gene duplication that predates the speciation event leading to divergence of C. difficile and P. sordellii. (B) Representative crystal structures and domain architectures are shown for TcdA (above) and TcdB (below). The structure of TcdA lacks the CROPS domain and is derived from PDB ID 4F04. The full-length structure of TcdB is based on PDB ID (6OQ5), and was modified to remove bound antibodies. Domain definitions were derived from Aktories et al. [24]. (TIF) [file ppat.1009181.s001.tif]

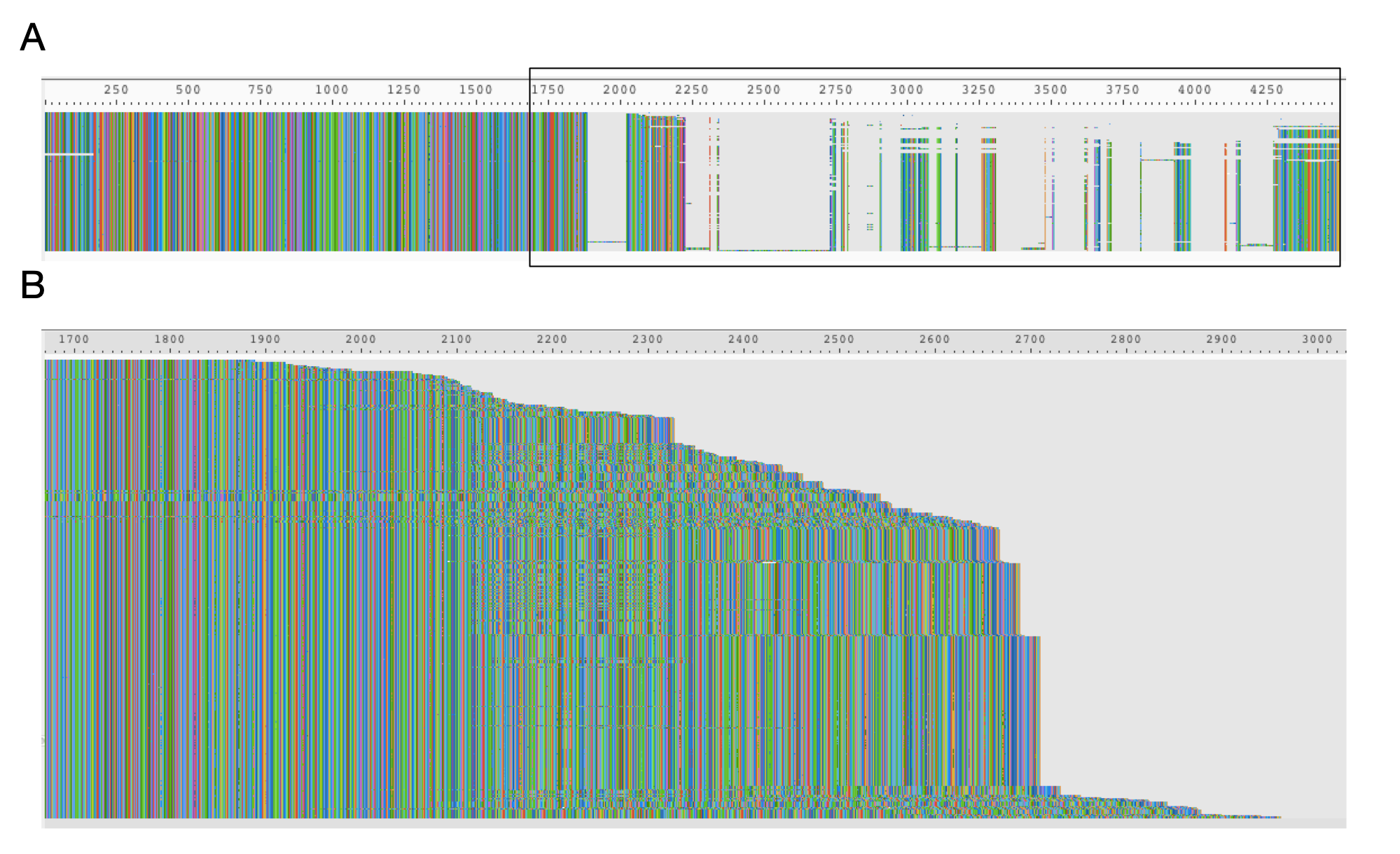

Supplement: S2 Fig — (A) Complete alignment of 480 unique TcdA sequences. (B) Visualization of unaligned sequences to display C-terminal length variation following residue ~900. (TIF) [file ppat.1009181.s002.tif]

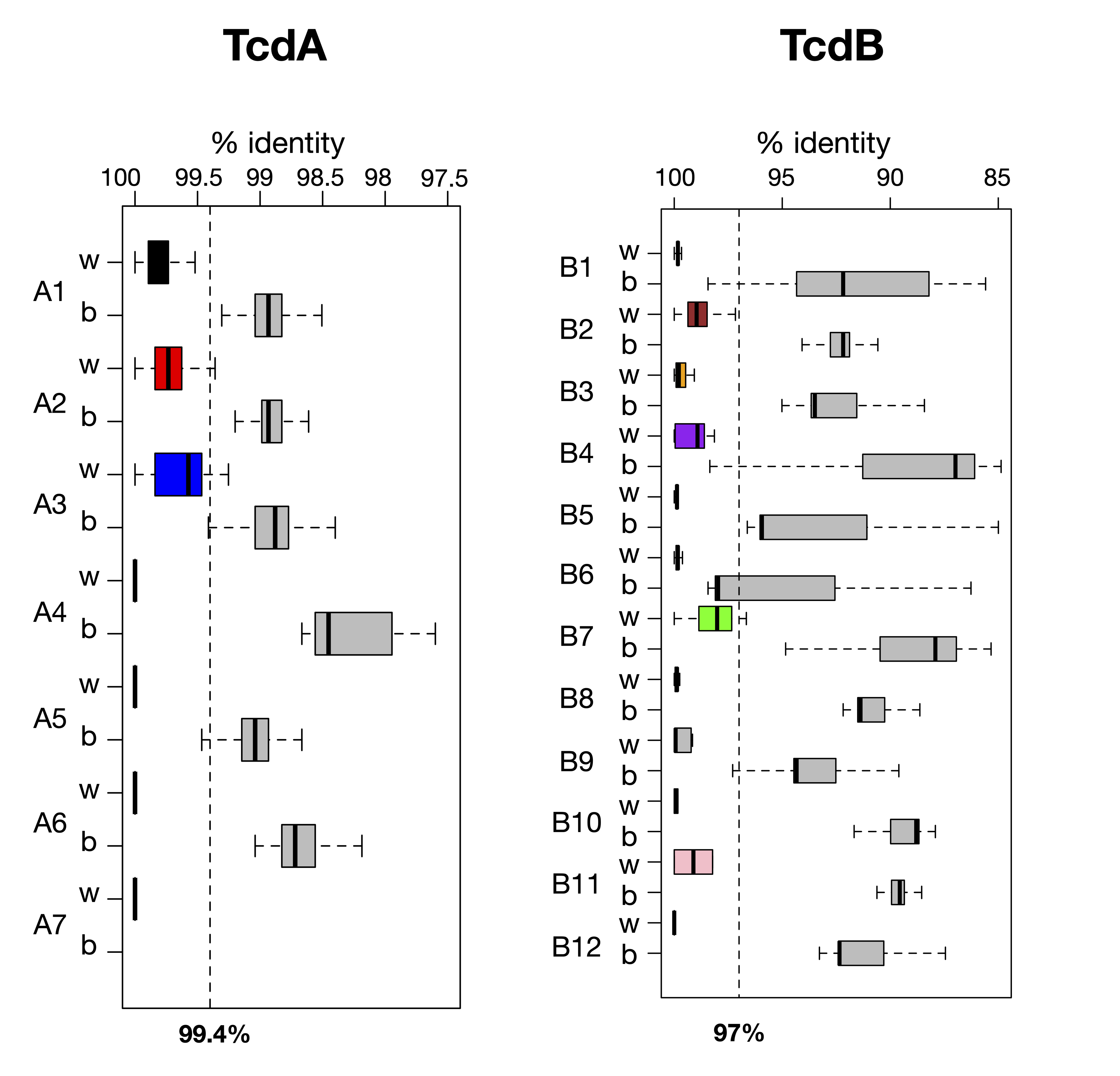

Supplement: S3 Fig — Pairwise sequence identities were calculated between all TcdA and TcdB sequences. The percentage identity distributions are plotted for sequences within (“w”) the same subtype versus between (“b”) subtypes for TcdA (left) and TcdB (right). As expected, the % identities are much higher within than between subtypes. For TcdA, a percentage identity threshold of 99.4 effectively distinguishes sequences within the same subtype, whereas for TcdB, a threshold of 97% effectively distinguishes sequences within the same subtype. (TIF) [file ppat.1009181.s003.tif]

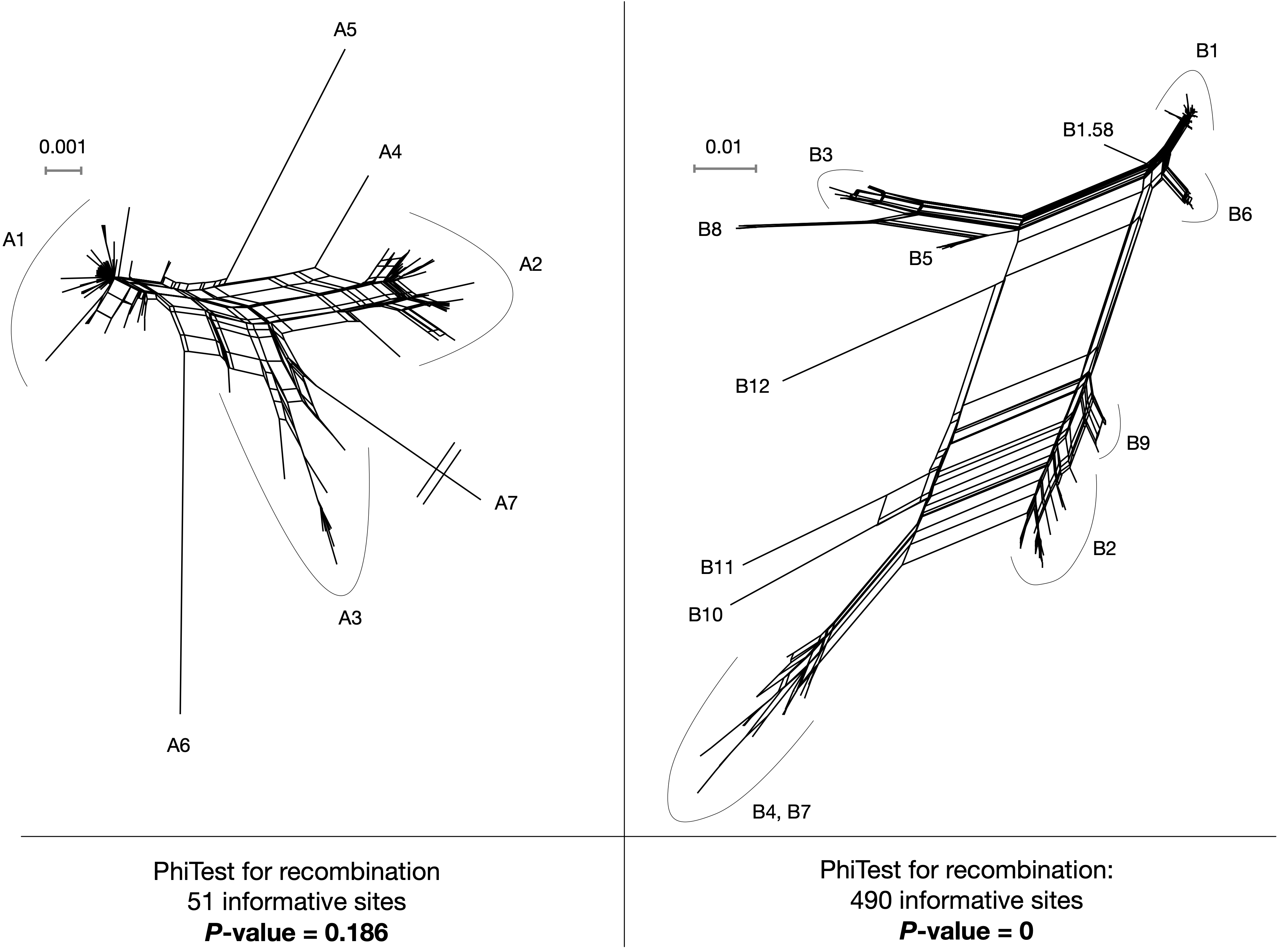

Supplement: S4 Fig — Split networks of TcdA and TcdB were generated using the SplitsTree software. Parallel edges suggest the existence of sites that are not compatible with a perfect monophyletic tree, which can result from recombination. An extremely long branch (A7) has been truncated in order to permit visualization. (TIF) [file ppat.1009181.s004.tif]

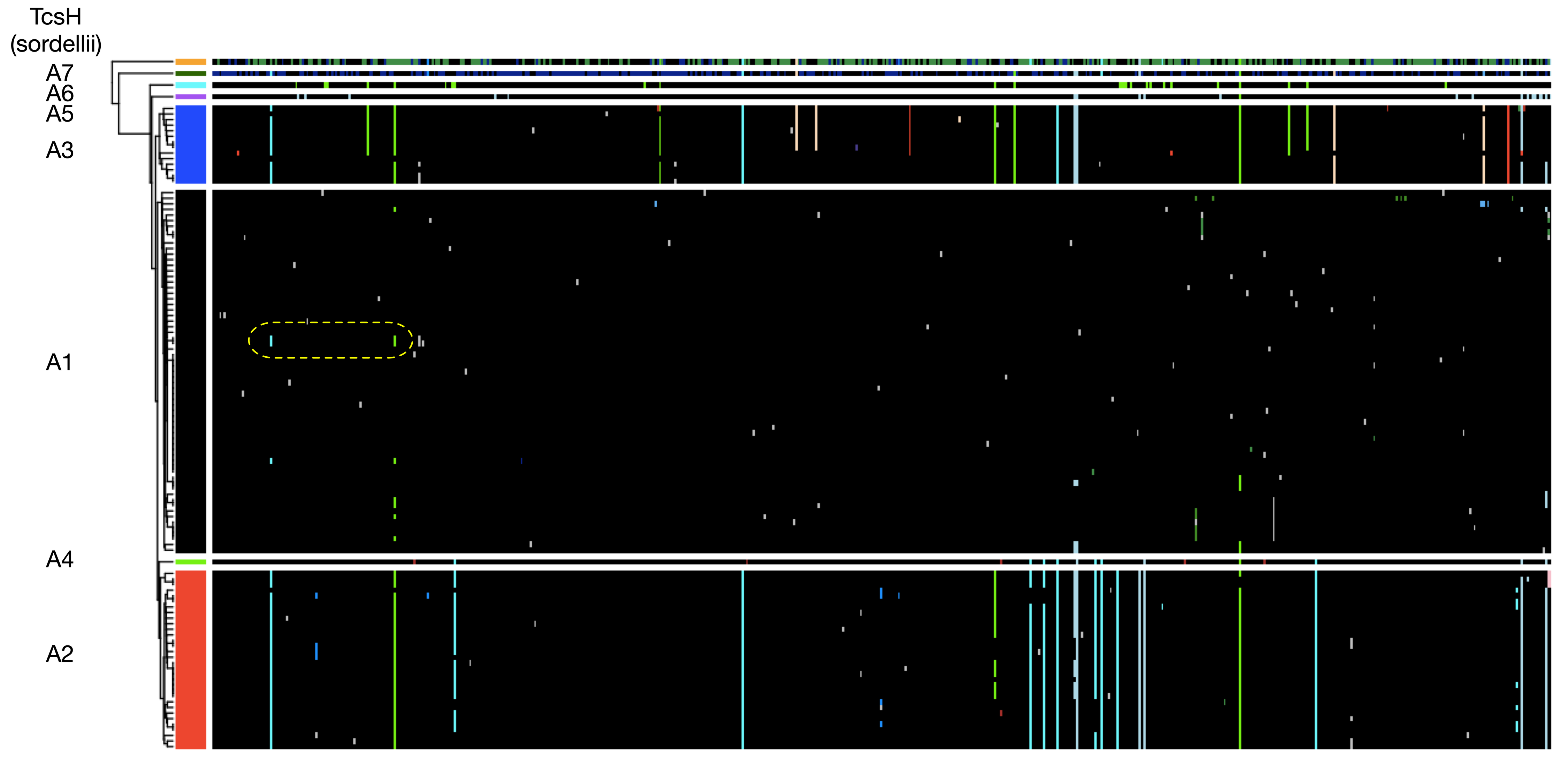

Supplement: S5 Fig — Visualization and analysis of amino acid variation patterns was performed using the HaploColor algorithm (https://github.com/doxeylab/haploColor), which was run for 16 iterations. Patterns of amino acid variation within each subtype are highly homogeneous, and thus a lack of evidence for recombination. One potential exception is highlighted in yellow, involving two amino acid variants that occur within subtype A1 that are lacking in most other A1 sequences but present in subtypes A2 and A3. However, this pattern may also be due to ancestral variation rather than recombination. Overall, compared to TcdB, the TcdA displays considerably less sequence variation and lacks the mosaic patterns that would result from recombination. (TIF) [file ppat.1009181.s005.tif]

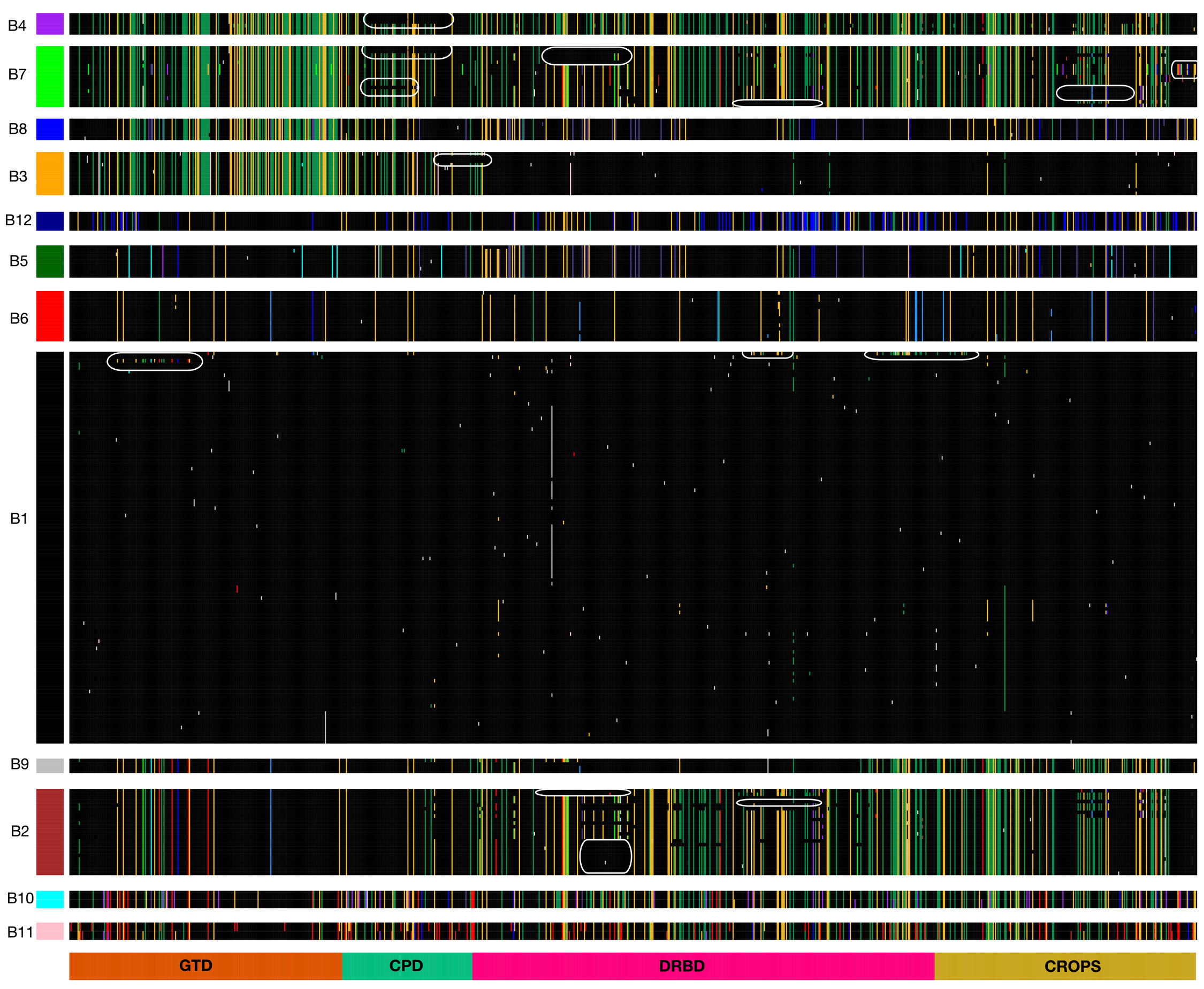

Supplement: S6 Fig — The TcdB multiple sequence alignment was colored using the HaploColor algorithm (https://github.com/doxeylab/haploColor), which was run for 16 iterations. Fourteen example segments containing amino acid variants that are unexpected for their subtype are shown by white ovals. These represent putative between-subtype microrecombination events. These fourteen are not a complete list as many more can be seen visually. (TIF) [file ppat.1009181.s006.tif]

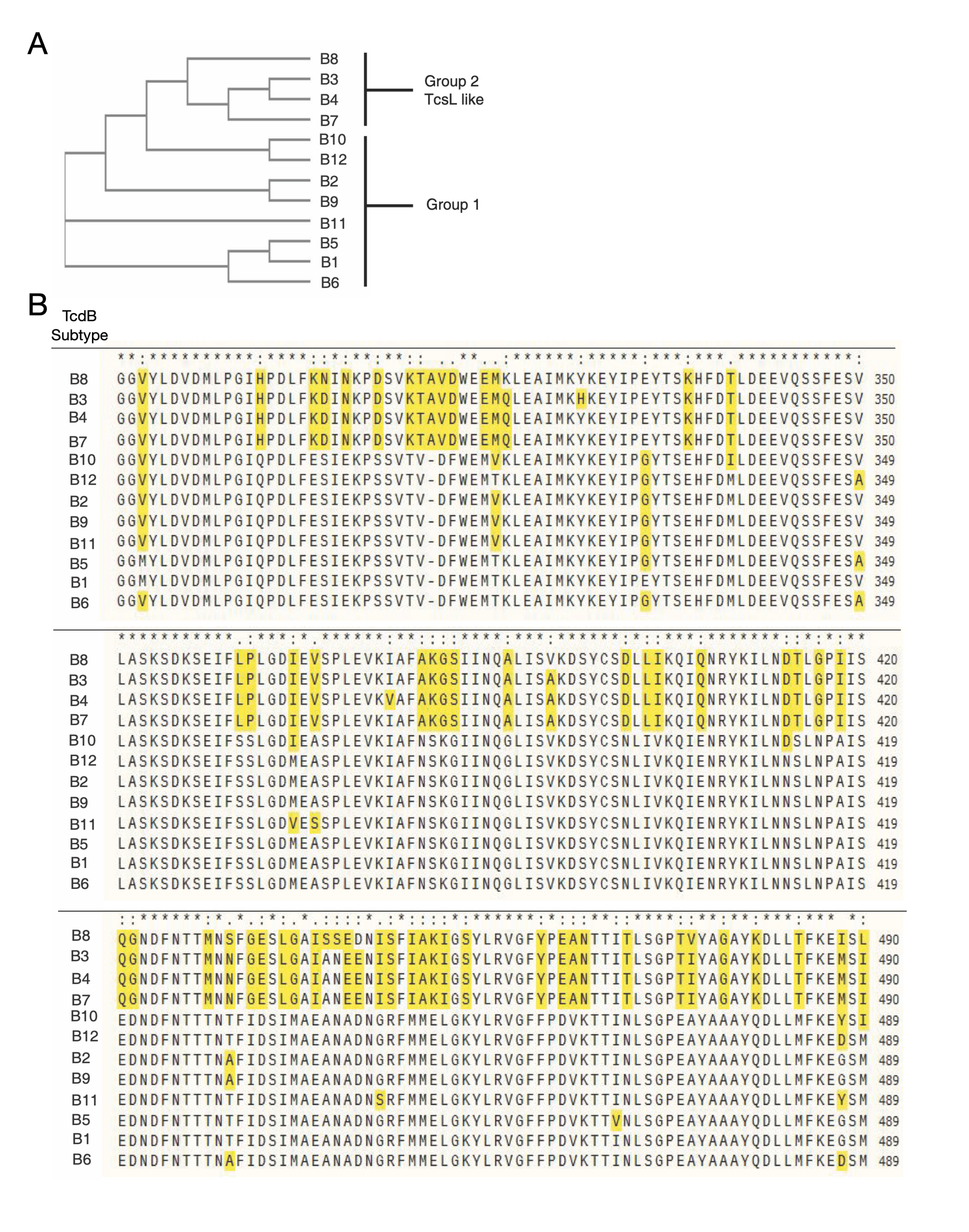

Supplement: S7 Fig — (A) Phylogenetic tree of the GTD domains. (B) alignment of region 280–490. According to the tree and the alignment, and the functions, GTD domains could be classed into two groups, one group is TcsL-like which gives vero cells rounding and clumping phenotypes (strain 1470 and 8846); the other group is the classical TcdB-like group which only give the rounding phenotype. (TIF) [file ppat.1009181.s007.tif]

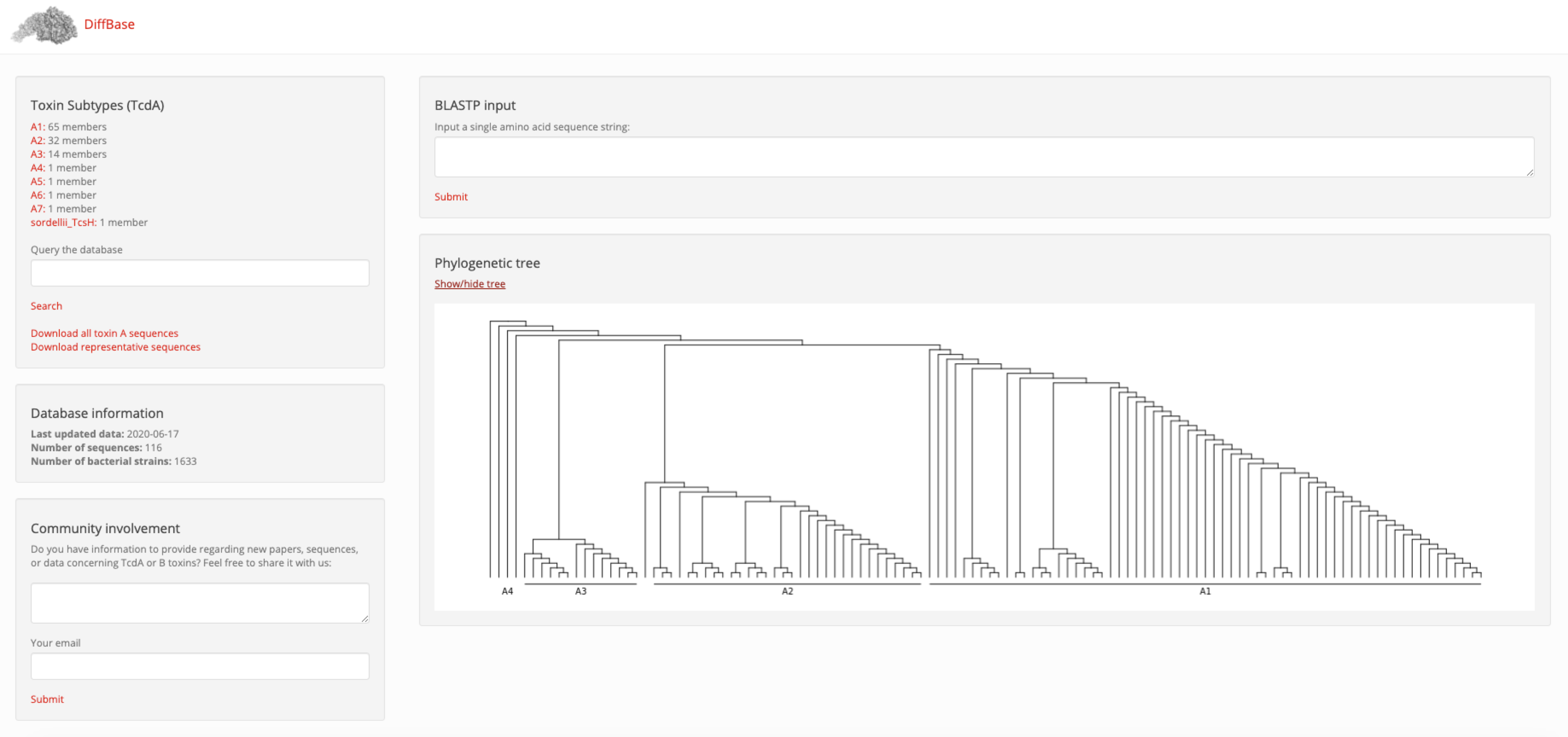

Supplement: S8 Fig — DiffBase is currently subdivided into two main sections for TcdA and TcdB sequences. Shown above is the TcdA portion of the database. (TIF) [file ppat.1009181.s008.tif]
