# Supplementary material for: Phylogenomics of 8,839 Clostridioides difficile genomes reveals recombination-driven evolution and diversification of toxin A and B
Source: PLoS Pathog. 2020 Dec 28;16(12):e1009181. doi: 10.1371/journal.ppat.1009181 (PMC7853461; doi:10.1371/journal.ppat.1009181)
Supplement: S1 Table — Novel sequences contain at least one substitution not observed in existing sequences derived from NCBI GenBank. (DOCX) [file ppat.1009181.s009.docx]

**S1 Table.** Subtypes of novel TcdA and TcdB sequences identified in the NCBI short read archive. Novel sequences contain at least one substitution not observed in existing sequences derived from NCBI GenBank.

| **Subtype** | **#** |
| --- | --- |
| A1 | 25 |
| A2 | 10 |
| A3 | 3 |
| A4 | 1 |
| A5 | 1 |
| A6 | 1 |
|  |  |
| B1 | 52 |
| B2 | 12 |
| B3 | 1 |
| B4 | 1 |
| B5 | 2 |
| B6 | 2 |
| B7 | 7 |
| B8 | 4 |
| B9 | 3 |
