# Supplementary material for: Phylogenomics of 8,839 Clostridioides difficile genomes reveals recombination-driven evolution and diversification of toxin A and B
Source: PLoS Pathog. 2020 Dec 28;16(12):e1009181. doi: 10.1371/journal.ppat.1009181 (PMC7853461; doi:10.1371/journal.ppat.1009181)
Supplement: S2 Table — Different groups are assigned unique colors. The table is based on information compiled from Rupnik and Janezic [46], Bletz et al. [12], and NCBI genome metadata. (DOCX) [file ppat.1009181.s010.docx]

**S2 Table.** List of clinically relevant and previously studied *C. difficile* strains, associated toxin subtypes, and toxinotypes. Different groups are assigned unique colors. The table is based on information compiled from Rupnik and Janezic [46], Bletz *et al*. [12], and NCBI genome metadata.

| **Toxinotype** | **Strain** | **Ribotype** | **Clade** | **TcdA subtype** | **TcdB subtype** | **Combined subtype** | **Toxin Production** |  |
| --- | --- | --- | --- | --- | --- | --- | --- | --- |
| 0 | VPI 10463 | 003/087 |  | A1.1 | B1.1 | A1/B1 | A+B+CDT− |  |
| 0 | 630 | 012 | 1 | A1.4 | B1.1 | A1/B1 |  |  |
| 0 | E28 | 012 |  | A1.38 | B1.1 | A1/B1 |  |  |
| 0 | T3 | 012 |  | A1.4 | B1.1 | A1/B1 |  |  |
| 0 | E14 | 014/020 |  | A1.1 | B1.2 | A1/B1 |  |  |
| 0 | CD166 | 014/020 |  | A1.1 | B1.5 | A1/B1 |  |  |
|  | CD111 | 014/020 |  | A1.1 | B1.2 | A1/B1 |  |  |
|  | CD109 | 014/020 |  | A1.1 | B1.2 | A1/B1 |  |  |
|  | CD90 | 014/020 |  | A1.1 | B1.2 | A1/B1 |  |  |
| 0 | CD43 | 027 |  |  | B1.5 | -/B1 |  |  |
| 0 | E12 | 106 |  | A1.1 | B1.4 | A1/B1 |  |  |
| 0 | 5555-DH/ST42 | 002 |  | A1.1 | B1.46 | A1/B1 |  |  |
| 0 | CD002 | 002 |  |  | B5.1 | -/B5 |  |  |
| 0/V | 597B | 131 |  | A1.14 | B1.56 | A1/B1 | A+B+CDT+ |  |
| I | EX 623 | 102 | 1 | A1.1 | B1.109 | A1/B1 | A+B+CDT+ |  |
| II | AC 008 | 103 | 1 | A1.1 | B1.3 | A1/B1 | A+B+CDT+ |  |
| III | R20291 | 027 | 2 | A2.1 | B2.1 | A2/B2 |  |  |
| IIIb | R 12087 (=CD196) | 027 | 2 | A2.1 | B2.1 | A2/B2 | A+B+CDT+ |  |
| IIIa | SE 844 | 080 | 2 | A2.5 | B9.1 | A2/B9 | A+B+CDT+ |  |
| IIIc | CH6230 | 251 | 2 | A2.7 | B2.3 | A2/B2 | A+B+CDT+ |  |
| IIIe | AI 541 | 251 | 2 | A2 | B2.7 | A2/B2 | A-B+CDT+ |  |
| IIId | 3073 | SLO 042 | 2 | A2.11 | B2.24 | A2/B2 | A+B+CDT+ |  |
| IV | 55767 | 023 | 3 | A3.2 | B6.1 | A3/B6 | A+B+CDT+ |  |
| V | SE 881 | 045 | 5 | A3.4 | B5 | A3/B5 | A+B+CDT+ |  |
| V | M120 | 078 | 5 | A3.1 | B5.1 | A3/B5 |  |  |
|  | NAP07 | 078 |  | A3.1 | B5.1 | A3/B5 |  |  |
| VI | 51377 | 127 | 5 | A3.1 | B5.1 | A3/B5 | A+B+CDT+ |  |
| VII | 57267 | 063 | 5 | A3.1 | B5.7 | A3/B5 | A+B+CDT+ |  |
| VIII | 1470 | 017 | 4 |  | B3.1 | -/B3 | A−B+CDT− |  |
| VIII | M68 | 017 |  |  | B3.1 | -/B3 |  |  |
| VIII | E13 | 017 |  |  | B3.1 | -/B3 |  |  |
| IXa | 51680 | 019 | 2 | A2.2 | B7.1 | A2/B7 | A+B+CDT+ |  |
| Ixb | TFA/V20-1 | 244 | 2 | A2.6 | B7.2 | A2/B7 |  |  |
| IXc | 8785 | 109 | 5 | A2 | B7.5 | A2/B7 | A+B+CDT+ |  |
| IXd | 1732874 | SLO 228 | 2 | A2.8 | B7.13 | A2/B7 | A+B+CDT+ |  |
| Xa | 8864 | 036 / 591(CE) | 2 |  | B4.1 | -/B4 | A−B+CDT+ |  |
| Xb | J9965 | SLO 032 | 2 |  | B4.4 | -/B4 | A−B+CDT+ |  |
| XIa | IS 58 | 033 | 5 |  |  | -/- | A−B−CDT+ |  |
| XId | OCD 5/2 | 033 | 5 |  |  | -/- | A−B−CDT+ |  |
| XIb | R 11402 | 288 (CE) | 5 |  |  | -/- | A−B−CDT+ |  |
| XII | TFA/V14-10 | 153(CE) | 2 |  |  | -/- | A−B−CDT+ |  |
| XII | IS 25 | 258 | 1 | A1.1 | B1.10 | A1/B1 | A+B+CDT- |  |
| XIII | R 9367 | 070 | 1 |  | B1.2 | -/B1 |  |  |
| XIVa | R 10870 | 111 | 2 | A2 | B7.3 | A2/B7 | A+B+CDT+ |  |
| XIVb | R 9385 | 122 | 2 | A2.12 | B7.6 | A2/B7 | A+B+CDT+ |  |
| XVI | SUC36 | 078 | 5 | A3.8 | B5.6 | A3/B5 | A+B+CDT+ |  |
| XVIII | K095 | 014 | 1 | A1.1 | B1.105 | A1/B1 | A+B+CDT− |  |
| XIX | TR13 | 018 | 1 | A1.2 | B1.2 | A1/B1 | A+B+CDT− |  |
| XX | TR14 | SLO 005 | 1 | A1.1 | B1.16 | A1/B1 | A+B+CDT− |  |
| XXI | CH6223 | SLO 035 | 4 | A1.35 | B3.11 | A1/B3 | A+B+CDT− |  |
| XXII | CD07-468 | 027 | 2 | A1.36 | B2.1 | A1/B2 | A+B+CDT+ |  |
| XXV | 7325 | 027 | 2 | A2.1 | B2.1 | A2/B2 | A+B+CDT+ |  |
| XXVI | 7459 | 050 (CE) | 1 |  | B1.6 | -/B1 | A-B+CDT− |  |
| XXVII | KK2443/2006 | SLO 037 | 1 |  |  | -/- | A-B-CDT− |  |
| XXVIII | CD08-070 | 126 | 5 | A3.1 | B5.1 | A3/B5 | A+B+CDT+ |  |
| XXIX | CD07-140 | 001 | 1 | A1.1 | B1.2 | A1/B1 | A+B+CDT− |  |
|  | CD92 | 001 |  | A1.1 | B1.2 | A1/B1 |  |  |
| XXX | ES 130 | SLO 101 | 5 |  | B8.1 | -/B8 | A-B+CDT+ |  |
| XXXI | WA 151 | SLO 098 |  |  | B8.2 | -/B8 | A-B+CDT+ |  |
| XXXII | 173070 | 151(CE) | C-II |  | B12.1 | -/B12 | A-B+CDT- |  |
| XXXIII | 2402 | SLO 086 | 1 | A1.37 | B3.10 | A1/B3 | A+B+CDT- |  |
| XXXIV | CD10-055 | SLO 201 |  |  | B2.7 | -/B2 | A-B+CDT- |  |
| XXXIII | 2402 | SLO 086 |  | A1.37 | B3.10 | A1/B3 | A+B+CDT- |  |
|  | RA09-70 |  |  | A7.1 | N | A7/- | A+B-CDT- |  |
|  | CD160 |  |  |  | B11.1 |  |  |  |
|  | HMX-149 |  |  | N | B11.2 | -/B11 | A-B+CDT- |  |
|  | CD10-165 |  | C-I | N | B10.1 | -/B10 | A-B+CDT- |  |
|  | SA10-050 |  | C-I | N | B10.2 | -/B10 | A-B+CDT- |  |
|  | HSJD-312 |  |  | N | B7.9 | -/B7 | A-B+CDT+ |  |
|  | HMX152 |  |  | N | B7.9 | -/B7 | A-B+CDT+ |  |
